# Trajectories of Nicotine and Cannabis Vaping and Polyuse From Adolescence to Young Adulthood

**DOI:** 10.1001/jamanetworkopen.2020.19181

**Published:** 2020-10-06

**Authors:** H. Isabella Lanza, Jessica L. Barrington-Trimis, Rob McConnell, Junhan Cho, Jessica L. Braymiller, Evan A. Krueger, Adam M. Leventhal

**Affiliations:** 1 Department of Human Development, California State University, Long Beach; 2 Department of Preventive Medicine, University of Southern California, Los Angeles; 3 Department of Psychology, University of Southern California, Los Angeles; 4 University of Southern California Norris Comprehensive Cancer Center, Los Angeles

## Abstract

**Importance:**

Little is known about cannabis vaping trajectories across adolescence and young adulthood or the co-occurrence with nicotine vaping.

**Objective:**

To evaluate nicotine vaping and cannabis vaping trajectories from late adolescence to young adulthood (≥18 years of age) and the extent of polysubstance vaping.

**Design, Setting, and Participants:**

In this prospective cohort study, 5 surveys (including information on substance vaped) were completed at 10 high schools in the Los Angeles, California, metro area. Students were surveyed at 6-month intervals from fall of 11th grade (October to December 2015; wave 5) through spring of 12th grade (March to June 2017; wave 8) and again approximately 1 to 2 years after high school (October 2018 to October 2019; wave 9).

**Exposures:**

Past 30-day nicotine and cannabis vaping frequency across 5 waves.

**Main Outcomes and Measures:**

Self-reported frequency of nicotine vaping and cannabis vaping within the past 30 days across 5 time points from late adolescence to young adulthood. Trajectories were measured with these past 30-day use frequencies at each wave. Parallel growth mixture modeling estimated conditional probabilities of polysubstance vaping.

**Results:**

The analytic sample included 3322 participants with at least 1 time point of data (mean [SD] age, 16.50 [0.42] years at baseline; 1777 [53.5%] female; 1573 [47.4%] Hispanic or Latino). Growth mixture modeling identified the 5-trajectory model as optimal for both nicotine vaping and cannabis vaping. Trajectories for nicotine and cannabis vaping were similar (nonusers: 2246 [67.6%] nicotine, 2157 [64.9%] cannabis; infrequent users: 566 [17.0%] nicotine, 608 [18.3%] cannabis; moderate users: 167 [5.0%] nicotine, 233 [7.0%] cannabis; young adult–onset frequent users: 213 [6.4%] nicotine, 190 [5.7%] cannabis; adolescent-onset escalating frequent users: 131 [3.9%] nicotine, 134 [4.0%] cannabis). Males had greater odds of belonging to the adolescent-onset escalating frequent users nicotine (adjusted odds ratio, 2.88; 95% CI, 1.58-5.23; *P* < .01) and cannabis (adjusted odds ratio, 1.95; 95% CI,1.03-3.66; *P* < .05) vaping trajectories compared with nonusers. Polysubstance vaping was common, with those in trajectories reflecting more frequent nicotine vaping (adolescent-onset escalating frequent users and young adult–onset frequent users) having a high probability of membership (85% and 93%, respectively) in a cannabis-use trajectory.

**Conclusions and Relevance:**

In this cohort study, the prevalence and type of nicotine vaping and cannabis vaping developmental trajectories from late adolescence to young adulthood were similar. Polysubstance vaping was common from late adolescence to young adulthood, particularly among those reporting more frequent vaping use. The findings suggest that public health policy and clinical interventions should address polysubstance vaping in both adolescence and young adulthood.

## Key Points

**Question:**

Do nicotine vaping and cannabis vaping follow similar developmental trajectories, and is polysubstance vaping common from late adolescence to young adulthood?

**Findings:**

In this cohort study of 3322 youths, developmental trajectories of nicotine vaping and cannabis vaping were similar and characterized by frequency and developmental timing of use. Youths in trajectories reflecting more frequent nicotine vaping had a high probability of membership in a cannabis-use trajectory.

**Meaning:**

In this study, polysubstance vaping was common from late adolescence to young adulthood particularly among those reporting more frequent vaping use.

## Teaser

This cohort study assesses the developmental trajectories of nicotine and cannabis vaping from late adolescence to young adulthood in Southern California.

## Introduction

The prevalence of electronic vaporizer use among US adolescents and young adults has substantially increased. Recent past 30-day estimates indicate marked increases for both nicotine vaping (20.9% to 25.4% among 12th graders from 2018 to 2019; 6.5% to 10.6% among young adults from 2017 to 2018) and cannabis vaping (7.5% to 14.0%% among 12th graders from 2018 to 2019; 6.6% to 9.3% among young adults from 2017 to 2018).^1,2,3^ There is a wide distribution in the frequency of past 30-day use among youths, ranging from vaping 1-2 days to daily use^4,5^; however, the extent to which this wide distribution represents individuals on escalating, deescalating, or stable use trajectories is unclear.

Growth mixture modeling (GMM) is a data-driven analytic approach for identifying unobserved subpopulations and describing distinct longitudinal change.^6,7^ This approach has been applied to identify youth trajectories of combustible cigarette, alcohol, and cannabis use.^8,9,10^ However, to date, only 2 longitudinal studies have sought to identify trajectories of nicotine vaping. Park et al^11^ identified 3 e-cigarette trajectories from 13 to 17 years of age: never (66.6%), low and increasing (20.1%), and high and increasing (13.3%). Westling et al^12^ reported 2 trajectories of e-cigarette use from 8th to 9th grade: 94.8% infrequent or no use and 5.1% accelerated use. Both studies indicated that membership in e-cigarette-using trajectories was associated with other substance use.

Although Park et al^11^ and Westling et al^12^ showed that adolescents can be classified into distinct trajectories of vaping, neither study examined the transition from adolescence to young adulthood. This is a population at high risk because transitions to college and/or the workforce and increased familial and financial responsibility are associated with increased risk of polysubstance use, enduring substance use problems, and substance use disorder.^13,14,15,16^ Furthermore, to our knowledge, developmental trajectories of cannabis vaping have not been identified in adolescence or young adulthood; thus, it is unknown how cannabis vaping develops over time. Examining gender and racial/ethnic differences of nicotine and cannabis vaping trajectories is also warranted because there is some evidence that males are more likely to vape than females^17,18^; racial/ethnic differences are less clear.^19,20^ In addition, although cross-sectional studies have reported high rates of nicotine and cannabis vaping polyuse among adolescents and young adults,^21,22,23,24^ to our knowledge, no longitudinal study has examined co-occurring development of nicotine and cannabis vaping trajectories. Identifying common polysubstance vaping patterns may inform both nicotine and cannabis policy and prevention.

 Using a prospective longitudinal design following a cohort of adolescents through young adulthood (≥18 years of age), the current study evaluated nicotine and cannabis vaping trajectories, demographic covariates of trajectories, and co-occurrence of nicotine and cannabis vaping trajectories.

## Methods

### Participants and Procedure

Data were drawn from a prospective cohort study of high school students from the Los Angeles, California, metro area. Students from 10 high schools were surveyed at 6-month intervals from 9th grade (October to December 2013; wave 1) through 12th grade (March to June 2017; wave 8) using self-administered pencil-and-paper questionnaires in schools. Participants were surveyed again 1 to 2 years after high school (October 2018 to October 2019) through online questionnaires. Questions pertaining to specific substance vaped were first asked at wave 5; thus, the current study assessed survey data from waves 5 to 9. All 9th graders at wave 1 were eligible to participate. This study was approved by the University of Southern California institutional review board. For waves 1 to 8, written or verbal parental consent and student assent were obtained. For wave 9, participants were contacted after turning 18 years of age and provided written informed consent. This study followed the Strengthening the Reporting of Observational Studies in Epidemiology (STROBE) and American Association for Public Opinion Research (AAPOR) reporting guidelines.

### Measures

#### Nicotine and Cannabis Vaping

Frequency of nicotine vaping and cannabis vaping was assessed across waves 5 to 9. Participants indicated the number of days of nicotine and cannabis vaping in the past 30 days by answering the following 2 questions. respectively: “In the last 30 days, how many total days have you used an electronic cigarette with nicotine (e-cigs, personal vaporizer, PV [personal vaporizer])?” “In the last 30 days, how many total days have you used an electronic device to vape THC [tetrahydrocannabinol] or hash oil (liquid pot, cannabis oil, weed pen, PAX Era [PAX Labs, Inc])?”. JUUL (JUUL Labs, Inc) use was also added to the wave 9 survey. Response categories (0, 1-2, 3-5, 6-9, 10-19, 20-29, or 30 days) were recoded into quantitative count variables by taking the median integer (rounded up when necessary) within each response range (0, 2, 4, 8, 15, 25, or 30 days), as done in previous work with this sample.^25,26^

#### Covariates

Age, highest parental educational level, gender, and race/ethnicity were self-reported. Highest parental educational level was recoded into a binary variable (college degree or higher vs some college or less). Race/ethnicity was recoded into 3 dummy variables (1, Asian; 0, non-Asian; 1, Hispanic or /Latino; 0, not Hispanic or Latino; 1, White; 0, non-White) for racial/ethnic groups representing 10% or more of the sample (Asian, Hispanic or Latino, and White).

### Statistical Analysis

Growth mixture modeling (GMM) was used to identify latent trajectories of nicotine and cannabis vaping. This approach captures heterogeneity within the population by identifying different growth trajectories of a latent variable based on unique intercepts and slopes.^27^ An increasing number of classes are estimated until an optimal model is identified. Similar to structural equation modeling in which statistical indexes (eg, confirmatory fit index, root mean square error of approximation) are used to identify the best-fitting model, the bayesian information criterion^28^ and Lo-Mendell-Rubin likelihood ratio test^29^ are commonly used indexes for GMM. Owing to the large number of 0s resulting in skewed distributions for past 30-day nicotine and cannabis vaping, vaping variables were treated as count outcomes; a zero-inflated Poisson model was used to account for both processes generating 0 days scores (vape users who did not use in the past 30 days and never users). Full information maximum likelihood was used to account for missing data, which allowed participants with at least 1 time point of data to be included in trajectory analyses (3322 of 3396 in the original cohort). Simulation studies report samples near 1200 as adequate for complex GMM.^30^ Covariates of each trajectory model were evaluated within the GMM framework using a validated 3-step approach to account for classification error.^31^ Parallel growth mixture modeling (PGMM) assessed co-occurrence of nicotine and cannabis vaping trajectories. PGMM estimates the unique developmental growth parameters of 2 distinct processes and how each process relates to the other across time.^32^ PGMM was used to calculate the probability of cross-classification between a specific nicotine vaping trajectory and cannabis vaping trajectory. A 2-sided *P* < .05 was considered to be statistically significant. Analyses were conducted with Mplus, version 8.4.^33^

## Results

### Descriptive Statistics

A total of 4100 students were eligible to enroll in the study; parental consent and student assent were obtained for 3396 adolescents (82.8%). Of the 3396 participants originally enrolled in the study, those with at least 1 time point of past 30-day vaping data from waves 5 to 9 were included in the analysis (n = 3322). Data distinguishing between nicotine vaping and cannabis vaping were available from waves 5 (fall of 11th grade) to wave 9 (young adulthood). Data were missing for 134 (4.0%) students in wave 5, 257 (7.7%) in wave 6, 179 (5.4%) in wave 7, 213 (6.4%) in wave 8, and 839 (25.3%) in wave 9.

Of the 3322 participants 1777 (53.5%) were female, and 1573 (47.4%) were Hispanic or Latino; the mean (SD) age at baseline was 16.50 (0.42) years (Table 1). From waves 5 to 8, the number of adolescents who reported any nicotine vaping in the past 30 days ranged from 139 (4.4%; in wave 7) to 232 (7.5%; in wave 8); the number then increased to 547 (22.0%) in wave 9 (young adulthood). For cannabis vaping, any use in the past 30 days steadily increased from 134 (4.2%) participants in wave 5 to 306 (9.8%) in wave 8 and then to 599 (24.1%) in wave 9. eTable 1 in the Supplement presents bootstrapped-derived correlations between nicotine and cannabis vaping and within-substance correlations. Across waves, nicotine vaping was significantly associated with cannabis vaping (*r*, 0.06-0.44; all *P* *<* .001 except for *P* = .002 for nicotine wave 9 and cannabis wave 5 and *P* = .01 for nicotine wave 5 and cannabis wave 9). Within substance correlations ranged from 0.22 to 0.55 for nicotine vaping and from 0.11 to 0.33 for cannabis vaping (*P* < .001 for all except *P* = .08 for cannabis in waves 5 and 9).

**Table 1.** Descriptive Characteristics

**Variable**	Participants (N = 3322)^a^
Age at baseline, mean (SD), y	16.50 (0.42)
Highest parental educational level	
College degree or higher	2014 (60.6)
Some college or less	856 (25.8)
Gender	
Female	1777 (53.5)
Male	1544 (46.5)
Race/ethnicity	
American Indian or Alaska Native	34 (1.0)
Asian	551 (16.6)
Black	161 (4.8)
Hispanic or Latino	1573 (47.4)
Native Hawaiian or Pacific Islander	138 (4.2)
White	533 (16.0)
Multiracial	216 (6.5)
Other	49 (1.5)
Unknown	67 (2.0)
Nicotine vaping, any past 30-d use	
Wave 5	
No/total No. (%)	183/3188 (5.7)
Mean (SD), d	8.50 (9.72)
Wave 6	
No/total No. (%)	167/3065 (5.4)
Mean (SD), d	8.53 (9.20)
Wave 7	
No/total No. (%)	139/3143 (4.4)
Mean (SD), d	8.65 (10.04)
Wave 8	
No/total No. (%)	232/3109 (7.5)
Mean (SD), d	9.38 (10.01)
Wave 9	
No/total No. (%)	547/2483 (22.0)
Mean (SD), d	11.34 (11.18)
Cannabis vaping, any past 30-d use	
Wave 5	
No/total No. (%)	134/3188 (4.2)
Mean (SD), d	8.87 (9.37)
Wave 6	
No/total No. (%)	137/3065 (4.7)
Mean (SD), d	8.96 (9.18)
Wave 7	
No/total No. (%)	176/3143 (5.6)
Mean (SD), d	8.93 (9.68)
Wave 8	
No/total No. (%)	306/3109 (9.8)
Mean (SD), d	8.61 (9.05)
Wave 9	
No/total No. (%)	599/2483 (24.1)
Mean (SD), d	9.78 (9.58)

^a^ Data are presented as number (percentage) of participants unless otherwise indicated. Baseline was wave 5, fall of 11th grade; wave 6, spring of 11th grade; wave 7, fall of 12th grade; wave 8, spring of 12th grade; and wave 9, young adulthood.

### Nicotine Vaping Trajectories

**Figure 1.**
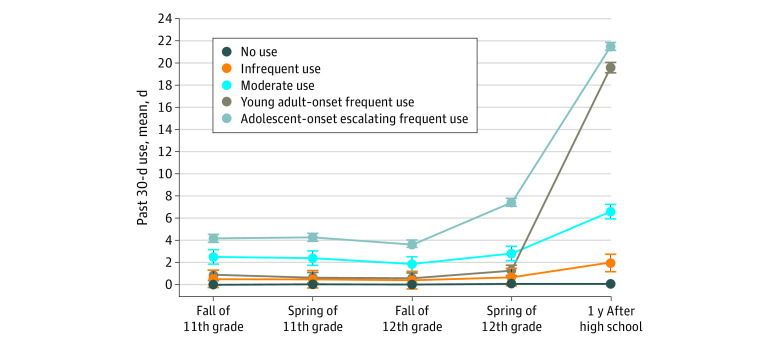
Growth Mixture Model of 5 Nicotine Vaping Trajectories Representing Past 30-Day Use of Nicotine Vaping During Adolescence and Young Adulthood Error bars indicate 95% CIs.

Model fit was evaluated across an increasing number of trajectory classes. The bayesian information criterion and Lo-Mendell-Rubin likelihood ratio test indicated that the 5-class model was the best fit (eTable 2 in the Supplement); the bayesian information criterion value was lowest for the 5-class model, and the Lo-Mendell-Rubin likelihood ratio test was not significant past the 5-class solution. Figure 1 presents mean days of past 30-day nicotine vaping at each wave for each identified trajectory. The largest trajectory class (non-users; 2246 [67.6%] of the sample) was composed of nonnicotine vapers across waves. The other 4 trajectories were characterized by varying nicotine vaping frequency and developmental timing. Infrequent users (566 [17.0%]) reported a mean of 0.45 days (95% CI, 0-1.22 days) of nicotine vaping in adolescence and a mean of 1.89 days (95% CI, 1.12-2.66 days) in young adulthood. Moderate users (167 [5.0%]) reported nicotine vaping for a mean of 2.33 days (95% CI, 1.67-2.99 days) in adolescence and a mean of 6.54 days (95% CI, 5.88-7.20 days) in young adulthood. Young adult–onset frequent users (213 [6.4%]) reported a mean of 0.76 days (95% CI, 0.29-1.23 days) of nicotine vaping in adolescence, with an increase to a mean of 19.75 days (95% CI, 19.28-20.22 days) in young adulthood. Adolescent-onset escalating frequent users (131 [3.9%]) were characterized by escalating frequent nicotine vaping use from late adolescence (mean, 7.38 days; 95% CI, 7.02-7.74 days) to young adulthood (mean, 21.49 days; 95% CI, 21.13-21.84 days).

### Cannabis Vaping Trajectories

**Figure 2.**
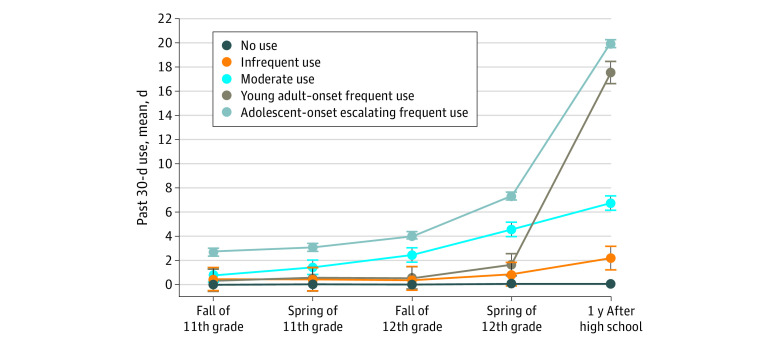
Growth Mixture Model of 5 Cannabis Vaping Trajectories Representing Past 30-Day Use of Cannabis Vaping During Adolescence and Young Adulthood Error bars indicate 95% CIs.

Similar to the nicotine vaping trajectories, the bayesian information criterion and Lo-Mendell-Rubin likelihood ratio test indicated that the 5-class model was optimal (eTable 2 in the Supplement). The identified trajectories for cannabis vaping were similar to those identified for nicotine vaping (Figure 2). The largest trajectory class (nonusers; 2157 [64.9%] of the sample) was composed of non–cannabis vapers across waves. The other 4 trajectories were characterized by varying frequency and developmental timing of use. Infrequent users (608 [18.3%]) reported a mean of 0.57 days (95% CI, 0-1.55 days) of cannabis vaping in adolescence and a mean of 2.19 days (95% CI, 1.21-3.17 days) in young adulthood. Moderate users (233 [7.0%]) reported cannabis vaping for a mean of 4.60 days (95% CI, 3.98-5.22 days) in adolescence and a mean of 6.75 days (95% CI, 6.13-7.37 days) in young adulthood. Young adult–onset frequent users (190 [5.7%]) reported little cannabis vaping in adolescence (mean, 0.74 days; 95% CI, 0-1.66 days) but an increase was observed in young adulthood (mean, 17.57 days; 95% CI, 16.65-18.49 days). Adolescent-onset escalating frequent users (134 [4.0%]) reported escalating frequent cannabis vaping use from late adolescence (mean, 7.31 days; 95% CI, 7.02-7.60 days) to young adulthood (mean, 19.94 days; 95% CI, 19.65-20.23 days).

### Covariates of Nicotine and Cannabis Vaping Trajectories

Covariates were added to each vaping trajectory model to assess the odds of trajectory membership based on age, highest parental educational level, gender, and race/ethnicity (Table 2). For nicotine vaping trajectories, males (vs females) had higher odds of membership in the adolescent-onset escalating frequent users (adjusted odds ratio [aOR], 2.88; 95% CI, 1.58-5.23; *P* = .001) and moderate users (aOR, 1.98; 95% CI, 1.26-3.14; *P* = .003) trajectories compared with nonusers. Latino and Hispanic individuals (vs non-Latino and non-Hispanic individuals) had lower odds of membership in the adolescent-onset escalating frequent users trajectory compared with the nonusers trajectory (aOR, 0.38; 95% CI, 0.17-0.82; *P* = .01). For cannabis vaping trajectories, males (vs females) had higher odds of membership in the adolescent-onset escalating frequent users compared with the nonusers trajectory (aOR, 1.95; 95% CI, 1.03-3.66; *P* = .04).

**Table 2.** Estimated Adjusted Odds Ratios of Nicotine Vaping and Cannabis Vaping Trajectory Membership

Characteristic	Odd Ratio (95% CI)^a^							
	Infrequent users		Moderate users		Young adult–onset frequent users		Adolescent-onset escalating frequent users	
	Nicotine	Cannabis	Nicotine	Cannabis	Nicotine	Cannabis	Nicotine	Cannabis
Age	0.83 (0.60-1.13)	1.00 (0.74-1.36)	0.87 (0.46-1.63)	0.86 (0.51-1.44)	1.47 (0.84-2.60)	0.56 (0.29-1.09)	1.16 (0.61-2.19)	0.98 (0.47-2.04)
Highest parental educational level^b^	1.17 (0.86-1.61)	1.15 (0.85-1.56)	1.26 (0.72-2.21)	1.26 (0.75-2.10)	1.59 (0.74-3.41)	0.97 (0.49-1.94)	0.89 (0.43-1.83)	0.86 (0.43-1.72)
Male vs female	0.97 (0.75-1.26)	0.82 (0.63-1.05)	1.98 (1.26-3.14)^c^	1.05 (0.71-1.55)	1.16 (0.72-1.87)	0.98 (0.57-1.70)	2.88 (1.58-5.23)^d^	1.95 (1.03-3.66)^e^
Asian vs non-Asian	0.66 (.41-1.05)	0.87 (0.58-1.31)	0.79 (0.40-1.56)	0.77 (0.38-1.56)	1.72 (0.81-3.68)	0.74 (0.27-1.99)	0.81 (0.33-1.97)	0.91 (0.32-2.63)
Latino or Hispanic vs non- Latino or non-Hispanic	0.81 (0.56-1.16)	0.86 (0.61-1.22)	0.55 (0.30-1.00)	1.07 (0.62-1.85)	0.57 (0.28-1.19)	1.02 (0.46-2.24)	0.38 (0.17-0.82)^f^	1.02 (0.41-2.52)
White vs non-White	1.23 (0.82-1.86)	1.05 (0.70-1.57)	0.85 (0.41-1.73)	1.33 (0.71-2.49)	2.01 (0.98-4.11)	1.29 (0.54-3.05)	1.50 (0.71-3.18)	1.67 (0.64-4.36)

^a^ The reference was the nonusers trajectory.

^b^ Highest parental educational level was college degree or higher vs some college or less.

^c^ *P* = .003.

^d^ *P* = .001.

^e^ *P* = .004.

^f^ *P* = .01.

### Co-occurring Nicotine and Cannabis Vaping Trajectories

PGMM was used to assess co-occurring trajectories of nicotine and cannabis vaping. Before examining conditional probabilities of membership in the cannabis vaping trajectories given membership in the nicotine vaping trajectories (Table 3), an overview of classification across both sets of trajectories indicated that 57.6% belonged to nonusers nicotine and cannabis vaping trajectories, 7.5% were classified into a nicotine-use but not a cannabis-use vaping trajectory, 9.8% were classified into a cannabis-use but not a nicotine-use vaping trajectory, and 25.1% belonged to both nicotine-use and cannabis-use vaping trajectories. As shown in Table 3, conditional probabilities indicated that participants in the nonusers nicotine vaping trajectory had the highest probability of membership in the nonusers cannabis vaping trajectory (85.5%). Of the nonusers nicotine vaping trajectory members who were members in one of the cannabis-use vaping trajectories (ie, single-substance cannabis vaping), most were in the infrequent users cannabis vaping trajectory (8.8%). For each of the 4 nicotine-use vaping trajectories, comembership in the nonusers cannabis vaping trajectory (ie, single-substance nicotine vaping) was uncommon (range, 6.7%-32.0%).

**Table 3.** Probability of Cannabis Vaping Trajectory Membership Based on Nicotine Vaping Trajectory Membership

Nicotine vaping	Cannabis vaping, %				
	Nonusers	Users			
		Infrequent	Moderate	Young adult-onset frequent	Adolescent-onset escalating frequent
Nonusers	85.5	8.8	1.4	3.8	0.4
Users					
Infrequent	32.0	38.7	15.3	9.1	4.8
Moderate	20.0	34.5	27.5	12.1	5.8
Young adult–onset frequent	6.7	39.6	25.3	18.8	9.7
Adolescent-onset escalating frequent	15.0	26.5	2.9	10.0	45.5

Participants in the infrequent users nicotine vaping trajectory had the highest probability of being classified in the infrequent users (38.7%) and nonusers (32.0%) cannabis vaping trajectories. Individuals in the moderate users nicotine vaping trajectory had higher probabilities of belonging to the infrequent users (34.5%) and moderate users (27.5%) cannabis vaping trajectories. Participants in the young adult–onset frequent users nicotine vaping trajectory had the highest probability of belonging to the infrequent users (39.6%) and moderate users (25.3%) cannabis vaping trajectories. Those in the adolescent-onset escalating frequent users nicotine vaping trajectory had the highest probability of membership in the adolescent-onset escalating frequent users cannabis vaping trajectory (45.5%).

## Discussion

The current study corroborates prior research showing associations between nicotine and cannabis vaping in adolescence.^21,22,23,24^ Our findings go beyond this literature by identifying developmental trajectories of cannabis vaping and examining co-occurring nicotine and cannabis vaping trajectories from late adolescence into young adulthood. Results indicate that trajectories of nicotine and cannabis vaping were similar; nonusers, infrequent users, moderate users, young adult–onset frequent users, and adolescent-onset escalating frequent users characterized vaping trajectories. Our co-occurring trajectory assessment allowed for the identification of distinct polysubstance vaping use and found that those in nicotine-use trajectories had a high likelihood of belonging to cannabis-use trajectories. Polysubstance vaping was common (25.1% of sample). For each nicotine-use vaping trajectory, the probability of being classified in a cannabis-use vaping trajectory ranged from 68% to 93% depending on the frequency of nicotine vaping. These findings suggest that addressing polysubstance vaping in public health policy and showing the potential benefits of prevention efforts in young adulthood in addition to adolescence are important.

For both nicotine and cannabis vaping, 5 trajectories distinguished between frequency and developmental timing of use, which is consistent with previous studies that identified adolescent e-cigarette trajectories characterized by frequency and increasing use.^11,12^ Of note, the proportion of the sample in each trajectory and type of trajectory were similar between nicotine and cannabis vaping. These similarities suggest that developmental trajectories of nicotine and cannabis vaping may share underlying risk processes, which has been observed in polyuse of combustible tobacco and cannabis use.^34,35^ In this study, males (vs females) had close to double (cannabis) and triple (nicotine) the odds of belonging to the adolescent-onset escalating frequent users trajectory compared with the nonusers trajectory. These results align with previous findings indicating that male adolescents have a higher likelihood of vaping than female adolescents^17,18^ and suggest that males who begin vaping in adolescence may be more likely than females to escalate their intake substantially. Race/ethnicity was not associated with membership in similar nicotine and cannabis vaping trajectories, but Latino individuals (vs non-Latino individuals) had lower odds of belonging to the adolescent-onset escalating frequent users nicotine trajectory than to the nonusers trajectory. As public health initiatives expand from their focus on nicotine vaping to other vaped substances, such as cannabis, understanding shared processes and pathways to polysubstance vaping is critical to appropriately tailor public health and clinical interventions to those most at risk.

The methods used to examine development of nicotine and cannabis vaping from late adolescence into young adulthood allowed for the identification of a trajectory (young adult–onset frequent users) that was characterized by frequent vaping in young adulthood but not in adolescence. The increase in both past 30-day nicotine and cannabis vaping observed among young adult–onset frequent users between the spring of 12th grade (mean, 1-2 days) and young adulthood (mean, 18-20 days) is noteworthy. Although a cohort effect may have been associated with increased use of nicotine and cannabis vaping during the young adulthood wave, which spanned from 2018 to 2019 when pod vaporizers, such as JUUL, and disposable vaporizers increased in popularity and marijuana use in California was legalized,^36,37,38^ research is needed to elucidate why some individuals may have a greater risk of nicotine and/or cannabis vaping in young adulthood but not adolescence; these individuals may not be following the previously established adolescent pathways to vaping.^39,40,41^ A substantial number of young adults initiating nicotine or cannabis vaping may be overlooked by public health efforts focused on adolescent vaping. Although prevention strategies focused on adolescent vaping should remain prominent, efforts specifically addressing young adult vaping use may be warranted to substantially reduce nicotine and cannabis vaping.

By examining co-occurring nicotine and cannabis vaping trajectories, this study was able to show that most individuals classified into a nicotine-use vaping trajectory were also classified into a cannabis-use vaping trajectory. Those in trajectories reflecting more frequent nicotine vaping use (adolescent-onset escalating frequent users and young adult–onset frequent users) had a high probability of membership (85% and 93%, respectively) in a cannabis-use vaping trajectory. Although 46% of adolescent-onset escalating frequent users of nicotine vaping were classified into the corresponding cannabis vaping trajectory, young adult–onset frequent users had higher probabilities of belonging to the infrequent (40%) or moderate (25%) users cannabis vaping trajectories, which suggests the possibility of distinct risk processes and pathways to polysubstance vaping in adolescence vs young adulthood. Furthermore, policies targeting nicotine vaping through restrictions on flavors and cartridge-based devices^42^ may need to expand to other vaped substances, such as cannabis, to better address co-occurring health risk behaviors among younger populations. Addressing polysubstance vaping is critical because polysubstance vaping users are likely to be at high risk of deleterious health outcomes common among combustible tobacco and cannabis polyusers, such as cognitive deficits, mental health impairments, and greater substance dependence.^43,44,45,46^

### Limitations

This study has limitations. The use of a sample specific to Southern California limits generalizability to other regions; however, a regionally specific sample increases the likelihood that participants were experiencing similar regulatory policies and trends in vaping use. The study also relied on self-report of vaping use, but self-report is the most common method of measuring substance use. Although the sample was racially/ethnically diverse, evaluating racial/ethnic differences between Black individuals and other ethnicity/race participants was not feasible owing to low representation. There were more missing data in wave 9 (young adulthood) compared with waves 5 to 8 (adolescence); however, full information maximum likelihood enabled participants with at least 1 wave of data to be included in the analysis. Although the study examined the transition from adolescence to young adulthood, additional waves in young adulthood would better inform young adult vaping use.

## Conclusions

In this study, nicotine and cannabis vaping trajectory models were similar; this, additional work appears to be needed to evaluate shared risk processes. A significant proportion of individuals initiated and participated in both nicotine and cannabis vaping during young adulthood, suggesting that research is warranted to identify developmentally appropriate interventions. Further study of the substantial polysubstance vaping observed between nicotine and cannabis vaping trajectories appears to be needed to develop more effective regulatory practices and interventions.

## Notes

Supplement. **eTable 1.** Correlations between nicotine vaping and nicotine cannabis across waves **eTable 2.** Model fit indices for nicotine vaping trajectories
