# Supplementary material for: Trajectories of Nicotine and Cannabis Vaping and Polyuse From Adolescence to Young Adulthood
Source: JAMA Netw Open. 2020 Oct 6;3(10):e2019181. doi: 10.1001/jamanetworkopen.2020.19181 (PMC7539114; doi:10.1001/jamanetworkopen.2020.19181)
Supplement: Supplement. — eTable 1. Correlations between nicotine vaping and nicotine cannabis across waves eTable 2. Model fit indices for nicotine vaping trajectories [file jamanetwopen-e2019181-s001.pdf]

Supplemental Online Content

Lanza HI, Barrington-Trimis JL, McConnell R, et al. Trajectories of Nicotine and Cannabis Vaping and Polyuse From Adolescence to Young Adulthood. *JAMA Netw Open*. 2020;3(10):e2019181. doi:10.1001/jamanetworkopen.2020.19181

eTable 1. Correlations between nicotine vaping and nicotine cannabis across waves

eTable 2. Model fit indices for nicotine vaping trajectories

This supplemental material has been provided by the authors to give readers additional information about their work.

© 2020 Lanza HI et al. *JAMA Network Open.*

eTable 1. Correlations between nicotine vaping and nicotine cannabis across waves

	Nicotine W5	Nicotine W6	Nicotine W7	Nicotine W8	Nicotine W9	Cannabis W5	Cannabis W6	Cannabis W7	Cannabis W8	Cannabis W9
Nicotine W5						.42	.17	.17	.17	.08
Nicotine W6	.42					.24	.36	.14	.19	.14
Nicotine W7	.44	.52				.16	.13	.32	.11	.09
Nicotine W8	.36	.49	.53			.18	.17	.10	.30	.12
Nicotine W9	.21	.21	.22	.26		.10	.12	.11	.17	.34
Cannabis W5										
Cannabis W6						.44				
Cannabis W7						.29	.36			
Cannabis W8						.33	.42	.30		
Cannabis W9						.10	.19	.18	.26	

W5: Wave 1 – fall 11th grade; W6: Wave 2 – spring 11th grade; W7: Wave 3 – fall 12th grade; W8: Wave 4 – spring of 12th grade; W9: Wave 9 – young adulthood.

All correlations significant at $p < .01$.

© 2020 Lanza HI et al. *JAMA Network Open.*

**eTable 2.** Model fit indices for nicotine vaping trajectories							
Trajectories	Log Likelihood	Free Parameters	BIC[a]	Adjusted BIC[b]	AIC[c]	LMR LRT *p*-value for *k*-1[d]	Entropy
1	-12460.22	8	24985.30	24959.88	24936.43	N/A	N/A
2	-9174.33	12	18445.95	18407.82	18372.65	< .001	.31
3	-8459.55	16	17048.83	16997.99	16951.11	<.01	.30
4	-8171.14	20	16504.44	16440.89	16382.28	.22	.31
5	-7791.94	24	15778.47	15702.22	15631.88	<.001	.77
6	-7668.79	28	15804.61	15675.64	15603.58	.09	.77
Model fit indices for cannabis vaping trajectories							
Trajectories	Log Likelihood	Free Parameters	BIC[a]	Adjusted BIC	AIC[b]	LMR LRT[c] *p*-value for *k*-1[d]	Entropy
1	-12195.19	8	24455.23	24429.81	24406.37	N/A	N/A
2	-9358.75	12	18814.79	18776.66	18741.50	< .001	.35
3	-8870.29	16	17870.29	17819.45	17772.58	.08	.33
4	-8508.47	20	17179.08	17115.53	17056.94	< .001	.74
5	-8254.20	24	16702.97	16626.72	16556.40	.04	.74
6	-8155.05	28	16737.11	16548.14	16516.10	.20	.74
[a]BIC = Bayesian information criterion; [b] sample-size adjusted Bayesian information criterion; [c]AIC = Akaike information criterion; [d]LMR LRT = Lo-Mendell-Rubin likelihood ratio test, *p*-value for *k*-1 refers to significant improvement in model fit between the class (*k*) and the class preceding it (*k*-1).							

© 2020 Lanza HI et al. *JAMA Network Open.*
